# Antibiotic Resistance to Molecules Commonly Prescribed for the Treatment of Antibiotic-Resistant Gram-Positive Pathogens: What Is Relevant for the Clinician?

**DOI:** 10.3390/pathogens13010088

**Published:** 2024-01-19

**Authors:** Gianpiero Tebano, Irene Zaghi, Francesco Baldasso, Chiara Calgarini, Roberta Capozzi, Caterina Salvadori, Monica Cricca, Francesco Cristini

**Affiliations:** 1 Infectious Diseases Unit, AUSL Romagna, Ravenna Hospital, 48121 Ravenna, Italy; irene.zaghi@auslromagna.it (I.Z.); chiara.calgarini@studio.unibo.it (C.C.); caterina.salvadori@auslromagna.it (C.S.); 2 Unit of Microbiology, The Greater Romagna Area Hub Laboratory, 47522 Cesena, Italy; monica.cricca3@unibo.it; 3 Infectious Diseases Unit, AUSL Romagna, Forlì and Cesena Hospitals, 47121 Forlì and Cesena, Italy; francesco.baldasso@studio.unibo.it (F.B.); roberta.capozzi@studio.unibo.it (R.C.); francesco.cristini@auslromagna.it (F.C.); 4 Department of Medical and Surgical Sciences (DIMEC), Alma Mater Studiorum, University of Bologna, 40126 Bologna, Italy

**Keywords:** Gram-positive bacterial infections, antibiotic resistance, daptomycin, dalbavancin, linezolid, tedizolid, ceftaroline, ceftobiprole, fosfomycin

## Abstract

Antibiotic resistance in Gram-positive pathogens is a relevant concern, particularly in the hospital setting. Several antibiotics are now available to treat these drug-resistant pathogens, such as daptomycin, dalbavancin, linezolid, tedizolid, ceftaroline, ceftobiprole, and fosfomycin. However, antibiotic resistance can also affect these newer molecules. Overall, this is not a frequent phenomenon, but it is a growing concern in some settings and can compromise the effectiveness of these molecules, leaving few therapeutic options. We reviewed the available evidence about the epidemiology of antibiotic resistance to these antibiotics and the main molecular mechanisms of resistance, particularly methicillin-resistant *Sthaphylococcus aureus*, methicillin-resistant coagulase-negative staphylococci, vancomycin-resistant *Enterococcus faecium*, and penicillin-resistant *Streptococcus pneumoniae*. We discussed the interpretation of susceptibility tests when minimum inhibitory concentrations are not available. We focused on the risk of the emergence of resistance during treatment, particularly for daptomycin and fosfomycin, and we discussed the strategies that can be implemented to reduce this phenomenon, which can lead to clinical failure despite appropriate antibiotic treatment. The judicious use of antibiotics, epidemiological surveillance, and infection control measures is essential to preserving the efficacy of these drugs.

## 1. Introduction

Antibiotic resistance is a widespread threat, causing significant morbidity and mortality [1]. Among Gram-positive antibiotic-resistant pathogens, methicillin-resistant *Staphylococcus aureus* (MRSA) [2], methicillin-resistant coagulase-negative staphylococci (MR-CoNS) [3], and vancomycin-resistant *Enterococcus faecium* (VRE) [4] are particularly relevant in the hospital setting. They are responsible for a wide range of health care-associated infections, such as bloodstream infections (BSI), surgical site infections, bone and joint infections, including prosthetic joint infections (PJI), and pneumonia (particularly *S. aureus*) [5]. However, the problem of antibiotic resistance in Gram-positive bacteria is not limited to the hospital setting. A particularly worrying phenomenon is represented by the spread of *Streptococcus pneumoniae* with reduced susceptibility to penicillin, or penicillin resistance, causing difficult-to-treat community-acquired pneumonia (CAP), sepsis, and central nervous system (CNS) infections [6,7]. Another relevant pathogen is community-acquired MRSA (CA-MRSA), which has been widely described and is responsible for severe non-nosocomial MRSA infections [7]. 

The spread of these resistant pathogens can compromise the efficacy of first-line anti-Gram-positive agents, such as antistaphylococcal penicillins and cefazolin for *Staphylococcus* spp. and ampicillin for *Streptococcus* spp. and *Enterococcus* spp. Fortunately, in the last two decades several new antibiotics renewed the therapeutic arsenal against antibiotic-resistant Gram-positive pathogens. The most relevant of these are daptomycin [8], dalbavancin [9], linezolid [10], tedizolid [11], ceftaroline [12], ceftobiprole [13], and fosfomycin [14]. The availability of these drugs allowed clinicians to limit the use of vancomycin, which is burdened by greater adverse effects and lower efficacy [15]. 

Overall, antibiotic resistance to these second-line agents is infrequent [16]. This is an emerging phenomenon which has clinical relevance as resistance to these antibiotics may leave very few therapeutic options for the treatment of critically ill patients. Moreover, the emergence of resistance during treatment has been described for some of these antibiotics (mainly daptomycin and fosfomycin) [17,18,19]. This is particularly worrying because it can compromise an initial clinical response and significantly complicate subsequent therapeutic management. 

The aim of this review was to summarize the most clinically relevant information about antibiotic resistance to daptomycin, dalbavancin, linezolid, tedizolid, ceftaroline, ceftobiprole, and fosfomycin.

### 1.1. Daptomycin

Daptomycin is a naturally derived lipopeptide antibiotic. It is active against *Staphylococcus* spp. (including MRSA and MR-CoNS), *Enterococcus* spp. (including VRE), *S. pneumoniae*, other *Streptococcus* spp. (including the viridans group), *Corynebacterium* spp., and Gram-positive anaerobes [8].

Daptomycin was originally approved for the treatment of acute bacterial skin and skin structure infections. Indications were extended in 2006 to the treatment of *S. aureus* blood stream infections (BSI) and right-sided infective endocarditis (IE). The licensed dose was 4–6 mg/kg once daily. However, the use of daptomycin in real-life practice has significantly evolved, including other indications such as left-sided IE, osteoarticular infections, and prosthetic joint infections (Table 1) [8,20,21,22]. Furthermore, higher doses are suggested (8–12 mg/kg) due to pharmacokinetic/pharmacodynamic (PK/PD) and clinical considerations, particularly in MRSA and VRE infections [20,23,24,25,26,27,28,29].

The main mechanism of action involves the insertion of daptomycin into the bacterial membrane, where it affects overall membrane fluidity, causing a calcium-dependent depolarization of the cell membrane followed by bacterial death [30]. 

According to the European Committee on Antimicrobial Susceptibility Testing (EUCAST) Breakpoint tables for interpretation of minimum inhibitory concentrations (MICs) and zone diameters, Version 14.0 [31], the susceptibility breakpoint was set at 1 mg/L for *Staphylococcus* spp. and *Streptococcus* groups A, B, C, and G [31], while no breakpoints were established for S. *pneumoniae* and *Enterococcus* spp. due to insufficient evidence (see Table 2, also reporting breakpoints from CLSI [32]). However, enterococci are known to be naturally less sensitive than staphylococci and streptococci to daptomycin, and the epidemiological cut-off (ECOFF) was set at 4 mg/L for *E. faecalis* and 8 mg/L for *E. faecium*. It is also worth mentioning that the determination of daptomycin MICs needs to be performed in the presence of 50 mg/L of Ca^2+^ for the broth dilution method [31]. The E-test can also be used to determine susceptibility, although relevant discrepancies with broth dilution have been reported [33].

Resistance has been described, but it remains very rare in *Staphylococcus* spp. and *S. pneumoniae* (≤1% of clinical isolates in Europe) [16,34,35,36]. Therefore, according to EUCAST [31], every resistant strain detected (particularly for *S. aureus*) should be sent to a reference laboratory for confirmation. On the contrary, the emergence of resistance is more frequent and clinically relevant in viridans streptococci [16,17]. Concerning *Enterococcus* spp., the isolation of strains exceeding the ECOFF is very rare [16,34,35]. However, according to PK/PD evaluations, even a 10–12 mg/kg dose is probably insufficient to reliably treat *Enterococcus* spp. with an MIC at the upper end of the wild type-distributions (i.e., 4–8 mg/L). Therefore, clinical failure can occur, even in the absence of resistance mechanisms [26].

The mechanisms leading to daptomycin resistance are not completely understood. In *Staphylococcus* spp., they concern different pathways, mainly ending up in cell membrane remodelling, cell wall thickening and changes in cell surface charge, preventing daptomycin insertion. Additional mechanisms of resistance have been described in *Enterococcus* spp., interfering with membrane homeostasis, phospholipid metabolism or stress response [37,38,39,40,41]. Due to the central role of the cell wall for both daptomycin bactericidal action and the emergence of daptomycin resistance, complex cross-resistance phenomena have been described as well as antibacterial synergisms with other antibiotics that target the bacterial cell wall. First, in rare but well-described strains of *S. aureus* with intermediate resistance to vancomycin (VISA), there is a high prevalence of daptomycin resistance (up to 80%), regardless of previous exposure to daptomycin [33,40]. On the other hand, the development of resistance to vancomycin in MRSA strains has been described during daptomycin therapy as being mediated by multiple peptide resistance factor (MprF) [42]. Another example of cross-reduced susceptibility is between dalbavancin and daptomycin during dalbavancin treatment [40,43].

Cell surface alterations that confer reduced susceptibility to daptomycin have also been linked to an increased susceptibility of MRSA to other cell wall-targeting antibiotics, such as â-lactams (so-called “seesaw” effect) [40,44,45,46]. This phenomenon has also been described in MR-CoNS [47]. Another mechanism of collateral susceptibility has been described in vitro in daptomycin-resistant VRE, where the acquisition of resistance to daptomycin translates into fitness costs and down-regulation of vancomycin-resistance genes [48].

Resistance to daptomycin can emerge during the treatment course, and this may represent a clinically relevant issue. The emergence of resistance in *S. aureus* is associated with high inoculum (IE, deep-seated infections), persistent infection, use of low doses (<6 mg/kg), and the presence of resistance to other antibiotics targeting the bacterial membrane [18,39,49]. The emergence of resistance during treatment has also been demonstrated in other species, such as viridans group streptococci (VGS) and *Corynebacterium* spp. In viridans group streptococci, particularly the *Streptococcus mitis/oralis* subgroup, this phenomenon is described both in vitro and in vivo and is clinically relevant [17,50]. Concerning *Corynebacterium* spp., daptomycin resistance is rarely reported, and it is observed in patients receiving prolonged daptomycin therapy, as shown in [38].

Two main strategies can be considered when the risk of resistance development is a possible concern: prescribing high doses and using combination therapy. As already highlighted, the need for doses higher than those originally licensed is strongly supported by PK/PD data and clinical data in several different populations, including among ICU and cancer patients. High doses (≥8 mg/kg for *Staphylococcus* spp. and 10–12 mg/kg for *Enterococcus* spp.) ensure higher success rates and a reduced risk of resistance onset. They do not determine a relevant increase in the frequency of muscular toxicity or other severe side effects, which remain rare [20,23,24,25,26,27,28,29]. 

Concerning combination therapy for MRSA, it encompasses the aforementioned association of daptomycin with beta-lactams (mainly anti-staphylococcal penicillins or ceftaroline), as well as the association with fosfomycin [51,52,53,54]. Similarly (although the data are mostly exploratory), combinations of daptomycin plus ampicillin, ertapenem or ceftaroline have been proposed for VRE [55] and daptomycin plus ceftriaxone or gentamycin for streptococci, particularly the *Streptococcus mitis/oralis* subgroup [21,56]. An analysis of clinical effectiveness, the ability to prevent the emergence of resistance, and the safety outcomes of each of these combinations is beyond the scope of this paper. However, it is possible to assert that the use of daptomycin in combination can be considered as standard of care in the context of IE (regardless of the species involved) [21] and in the treatment of viridans streptococci, since in these cases the risk of the emergence of resistance is sufficiently concrete. In other circumstances, combination therapy should be taken into consideration in case of difficult-to-treat infections [15,21,50,51,52,57].

### 1.2. Dalbavancin

Dalbavancin is a long-acting parenteral lipoglycopeptide, characterised by long half-life and a broad spectrum activity against Gram-positive pathogens, including MRSA, MR-CoNS, *Streptococcus* spp., and vancomycin-susceptible *Enterococcus* spp. [9,58].

Dalbavancin has been licensed for acute bacterial skin and skin structure infections [59,60]. However, its use has evolved over time, both in terms of suggested dose schedule [59] and clinical indications [61]. Currently, it can be considered as part of the therapeutic armamentarium in the treatment of osteomyelitis, prosthetic joint infections and BSI, including IE (Table 1) [21,61,62,63,64,65].

The antibacterial activity of dalbavancin is due to its ability to bind to the D-alanyl-D-alanine terminus of cell wall peptidoglycan. In particular, the long lipophilic tail anchors dalbavancin to the bacterial membrane, which in turn keeps it close to the D-alanyl-D-alanine terminus, interfering with the cell wall synthesis [9,58].

According to EUCAST (Table 2), the susceptibility breakpoint was set at 0.125 mg/L for *Staphylococcus* spp., *Streptococcus* groups A, B, C, and G, and viridans group streptococci. On the contrary, breakpoints and ECOFFs have not been defined for *S. pneumoniae* and *Enterococcus* spp. due to insufficient evidence [31].

To perform susceptibility testing and MIC determination for dalbavancin, the addition of polysorbate-80 (optimal concentration of 0.002%) is required in broth microdilution systems. Agar dilution methods are not validated. However, according to EUCAST, strains susceptible to vancomycin can be reported as susceptible to dalbavancin as well [31].

The main mechanism of resistance to dalbavancin occurs due to the loss of affinity for substituted peptidoglycan precursors. These are encoded by the Van gene complexes, particularly VanA. Consequently, most VRE exhibit dalbavancin resistance, although VanB genotype VRE can remain dalbavancin-susceptible. Vancomycin-susceptible *Enterococcus* spp. remain susceptible to dalbavancin, as well [9,66,67].

In *S. aureus* dalbavancin, resistance is anecdotal [66,67,68] and probably mediated by different mechanisms compared to VRE [69]. EUCAST recommends sending any resistant strain to a reference laboratory for confirmation [31]. Resistance development during therapy has been demonstrated in an in vitro model [70], but remains very rare in clinical experience. In their comprehensive review of the real-world use of dalbavancin published in 2021, Gatti et al. [61] identified 4 reports of resistance development to dalbavancin (3 MRSA and 1 methicillin-susceptible *S. aureus*—MSSA), mainly in patients affected by IE (3/4). Notably, the overall risk of resistance development was very low, despite a relevant number of patients with difficult-to-treat infections being included in this study, namely 114 patients with IE and 387 patients with bone and joint infections [61]. Another case report of resistance development during therapy was published in 2022 [69]. In the case of CoNS, dalbavancin resistance rate is very low and susceptible isolates account for 97–99% of the overall number [67,68,71]. The emergence of resistance during treatment has been reported very rarely as well [72]. Dalbavancin susceptibility is almost always preserved in *Streptococcus* groups A, B, C, and G, and in VGS [67,68].

### 1.3. Linezolid and Tedizolid

Linezolid is a synthetic oxazolidinone that was approved in 2000 for the treatment of Gram-positive pathogens, including MRSA, methicillin-resistant CoNS and VRE. Its spectrum of activity also includes *S. pneumoniae*, *Streptococcus* groups A, B, C, and G, viridans group streptococci, *Corynebacterium* spp., *Listeria monocytogenes*, and anaerobic Gram-positive bacteria [10,73,74]. It is widely used in clinical practice, mainly in acute bacterial skin and skin structure infections, pneumonia, osteoarticular infections, including PJI, and CNS infections [10,73,74]. It is also active against *Mycobacterium tuberculosis* and other *Mycobacteria* (Table 1) [75]. It exerts a bacteriostatic effect that inhibits protein synthesis by binding to a site on the bacterial 23S ribosomal RNA (rRNA) of the 50S subunit [10,73,74].

EUCAST (Table 2) has established susceptibility breakpoints at 4 mg/L for *Staphylococcus* spp. and *Enterococcus* spp. and at 2 mg/L for *S. pneumoniae*, *Streptococcus* groups A, B, C, and G, *Cutibacterium acnes*, *Corynebacterium* spp., and *Bacillus* spp. Breakpoints have not been established for VGS and *Listeria monocytogenes* [31]. ECOFFs, when available, are substantially superimposable with susceptibility breakpoints [16].

Resistance to linezolid is mediated primarily by multiple mutations in the 23S rRNA gene; other possible mechanisms are changes in the L3/L4 ribosomal proteins, and methylation of the 23S rRNA by a methylase designated as Cfr (chloramphenicol-florfenicol resistance). Resistance can be transmissible from other microorganisms via a mobile gene in the case of Cfr-mediated mechanisms, leading to clonal spreading and risk of outbreaks [76,77,78].

Overall, resistance has rarely been reported [79]. There are two main reasons for this. First, there is no significant cross-resistance, with other resistance mechanisms affecting other antibiotics targeting the protein synthesis [74]; second, most bacterial species have multiple 23S rRNA genes, and so resistance may require mutations in more than one of these genes to be clinically relevant (the so-called gene-dose effect) [39,80,81].

The first case of resistance to linezolid in *S. aureus* was reported in 2001 [82]. However, linezolid resistance remains very rare in *S. aureus* (including MRSA), affecting less than 0.5% of clinical isolates [16,81,83,84]. Resistance to linezolid may occur more frequently in CoNS, particularly *S. epidermidis*, although resistant strains remain less than 2% of the total [83]. Linezolid resistance has also been reported in VRE, but it is very rare in this pathogen as well, with reports from several countries signalling resistance in less than 1–2% of isolates [66,85,86]. Finally, linezolid resistance is anecdotal or not reported at all in *Streptococcus pneumoniae* [87], *Streptococcus* groups A, B, C, and G, VGS [88], and in other uncommonly isolated pathogens, including *Corynebacterium* spp. [89].

Long courses and repeated treatments seem to be the major risk factors for the development of resistance, as demonstrated for example in a heavily treated population of cystic fibrotic patients, where patients presenting linezolid-resistant *S. aureus* were subjected to a mean of 19 treatment courses with linezolid [90]. In rare cases of infections caused by linezolid-resistant *Staphylococcus* spp. or *Enterococcus* spp., and in absence of valid alternatives, some associations have been proposed, although they are supported only by exploratory and mostly in vitro data [91,92]. The use of higher doses, to the contrary, is generally not recommended, because of safety concerns [93].

Tedizolid is a once-daily oxazolidinone antibiotic that was approved in 2014 as noninferior to linezolid for the treatment of acute bacterial skin and skin structure infections. Its mechanism of action is similar to that of linezolid, and it works by binding to the 23S ribosomal RNA of the 50S subunit [11,94]. EUCAST (Table 2) has established a susceptibility breakpoint at 0.5 mg/L for *Staphylococcus* spp., *Streptococcus* groups A, B, C, and G, and *Streptococcus anginosus* group; moreover, for *Staphylococcus* spp. the susceptibility can be inferred from that for linezolid [31]. Overall, tedizolid resistance is very rare, similarly to linezolid resistance [95]. Cross-resistance with linezolid has been described, especially when a chromosomally mediated mechanism is involved. On the other hand, tedizolid is believed to retain activity against some linezolid-resistant isolates when the resistance mechanism is mediated by the plasmid-encoded Cfr gene [95,96].

### 1.4. Ceftaroline

Ceftaroline is a broad-spectrum cephalosporin, with potent antimicrobial activity against a wide range of Gram-positive and Gram-negative pathogens. The anti-Gram-positive spectrum includes *Staphylococcus* spp., *Streptococcus* spp. and *Micrococcus* spp., while activity against *Enterococcus* spp., *Listeria monocytogenes* and *Corynebacterium* spp. is moderate to poor [12,97,98].

As with other beta-lactam antibiotics, ceftaroline interferes with bacterial cell wall synthesis, causing cell death, by binding to penicillin-binding proteins (PBPs). Due to its high affinity for PBP2a (responsible for methicillin resistance in *Staphylococcus* spp.), ceftaroline was the first approved beta-lactam with preserved activity against MRSA and MR-CoNS [97,99].

Ceftaroline has been licensed for acute bacterial skin and skin structure infections and for community-acquired bacterial pneumonia, including cases with concomitant bacteraemia [97,100,101,102]. However, in real-life practice it has been used for several off-label indications, including IE and other intravascular infections, bone and joint infections, and diabetic foot infections (Table 1) [21,103,104,105].

EUCAST (Table 2) has established a susceptibility breakpoint at 1 mg/L for *Staphylococcus* spp. However, methicillin-susceptible strains can be reported as susceptible to ceftaroline. For *S. pneumoniae,* the susceptibility breakpoint is 0.25 mg/L, while for *Streptococcus* groups A, B, C, and G the susceptibility can be inferred from that for benzylpenicillin [31].

Resistance to ceftaroline has been reported in MRSA and MR-CoNS. It is associated with mutations that result in changes in PBP2a structure. Several amino-acid substitutions in PBP2a associated with ceftaroline resistance have been identified, both in non-penicillin-binding domains and penicillin-binding domains, with a cumulative effect in terms of increased MICs [106,107]. Multiple substitutions in PBPs have also been identified as the main mechanism of resistance in *S. pneumoniae* [108].

Data from international epidemiological reports showed that resistance among MRSA is infrequent, affecting less than 5–10% of isolates, although it can increase up to 25% in some settings [109,110,111]. Resistance has also been rarely reported in MR-CoNS. *S. haemolyticus* is by far the most affected [16,111]. On the other hand, ceftaroline susceptibility has been observed in extensively resistant strains, such as VISA and daptomycin-resistant and linezolid-resistant MRSA and CoNS [112].

Ceftaroline resistance in *S. pneumoniae* seems to remain very rare, ranging from 0% to <5% of isolates, even in settings with a high prevalence of multidrug-resistant (MDR) *S. pneumoniae* [108,110,112,113,114]. Finally, ceftaroline showed excellent activity, without resistance concerns, against *Streptococcus* spp. (including viridans group streptococci) and *Micrococcus* spp. [98].

The emergence of resistance to ceftaroline during treatment has been described but it appears to be of limited concern. It has been reported in case of difficult-to-treat infections, such as osteomyelitis and IE [115,116].

As already underlined, ceftaroline has been studied in combination with other anti-MRSA antibiotics (particularly daptomycin) for the treatment of BSI, including IE. This association has been proposed in order to increase the anti-bacterial efficacy and protect daptomycin from the emergence of resistance during treatment [15,21,51,52,57].

### 1.5. Ceftobiprole

Ceftobiprole is a broad-spectrum cephalosporin, showing antibacterial activity similar to that of ceftaroline and covering a wide range of Gram-positive and Gram-negative pathogens. Among Gram-positive pathogens, it is active against *Staphylococcus* spp., *Streptococcus* spp. and *Peptostreptococcus* spp. [13,117]. Moreover, ceftobiprole also has some activity against *E. faecalis*, although the MICs tend to be higher than those for *Staphylococcus* spp. and *Streptococcus* spp. [110].

Ceftobiprole has high binding affinity for PBP2a (conferring methicillin resistance in *S. aureus* and CoNS) and PBP2x (conferring penicillin resistance in *S. pneumoniae*). Therefore, it is active against MRSA, MR-CoNS and penicillin-resistant *S. pneumoniae* [13,110,117,118].

Ceftobiprole has been approved in the European Union and Canada for community-acquired and hospital-acquired pneumonia, excluding ventilator-associated pneumonia [119]. Off-label uses have been proposed, encompassing acute bacterial skin and skin structure infections. These also encompass BSI including IE, bone and joint infections, and mediastinitis (Table 1) [118,120,121,122,123].

EUCAST (Table 2) established a susceptibility breakpoint at 2 mg/L for *S. aureus* and gave indications about zone diameter breakpoints for CoNS. The ECOFF for *Staphylococcus* spp. is 1 mg/L. The strains which are susceptible to methicillin can be reported as susceptible to ceftobiprole for both *S. aureus* and CoNS. For *S. pneumoniae*, the susceptibility breakpoint is set at 0.5 mg/L. No breakpoints have been published for other Gram-positive species [31].

Ceftobiprole resistance has been described in MRSA and MR-CoNS, and it is associated with cumulative structural abnormalities in PBP2a [124]. Resistance has also been described in *S. pneumoniae*, and this is also due to PBP mutations [125].

However, ceftobiprole resistance remains rare in Gram-positive pathogens. In MRSA, resistant strains account for <2% of isolates in several international epidemiological reports [110,125,126,127,128]. One single-centre report showed a resistance rate of 12% [129]. In MR-CoNS, ceftobiprole resistance is found in less than 10% of strains; *S. haemolyticus* is particularly affected, as for ceftaroline [16,110,127]. Ceftobiprole resistance is also rare in *S. pneumoniae*, including MDR strains, and affects around <5% of isolates [110,125,128,130]. Finally, resistance is rare or anecdotal in other streptococci [118].

### 1.6. Fosfomycin

Fosfomycin has been introduced into clinical use for several decades, but for a long time it has been used mainly in the oral formulation for the treatment of uncomplicated urinary tract infections [14]. It is only recently that fosfomycin has been revalued for intravenous systemic use as it shows a broad-spectrum antibacterial activity, making it a possible alternative treatment for infections caused by MDR pathogens [131]. Regarding Gram-positive antibiotic-resistant bacteria, fosfomycin can retain activity against MRSA, MR-CoNS, VRE, and penicillin-resistant *S. pneumoniae* [132].

Intravenous fosfomycin is a low-molecular-weight, water-soluble compound with low-plasma protein binding. It is able to achieve significant serum and tissue concentrations, including of lung, cerebrospinal fluid, and bone [14,133,134]. It exerts antibacterial activity by blocking the synthesis of the bacterial wall at a step prior to that inhibited by β-lactams. It binds to the MurA enzyme, which is responsible for initiating the biosynthesis of peptidoglycan, leading to cell lysis [14,135]. Moreover, fosfomycin may reduce the adherence of bacteria to urinary epithelial cells [136] and to respiratory epithelial cells [137]. It is available in several European countries and Japan for the treatment of complicated urinary tract infections, respiratory tract infections, intra-abdominal infections, osteomyelitis, CNS infections, BSI, IE, and other intravascular infections (Table 1) [14,19,138,139].

EUCAST (Table 2) recently removed susceptibility breakpoint for *Staphylococcus* spp. Breakpoints have also not been set for other Gram-positive bacteria because of insufficient evidence. For *S. aureus* and enterococci, the ECOFF is provided and it is set at 32 mg/L and 128 mg/L, respectively. Agar dilution is considered the reference method for fosfomycin, and the determination of MICs requires the presence of 25 mg/L of glucose-6phosphate in the medium [31].

Intrinsic resistance to fosfomycin occurs mainly due to MurA mutations. Acquired resistance can be determined by modifications of membrane transporters, which prevents fosfomycin from entering the target cell, the acquisition of inactivating enzymes, and MurA mutations (less frequent). Some of the resistance determinants, particularly genes codifying for fosfomycin-inactivating enzymes, can be encoded in transferable plasmids, together with genes conferring resistance to other antibiotics [135]. The phenomenon of heteroresistance (presence of bacterial subpopulations with lower fosfomycin susceptibility) has been reported, particularly in *S. pneumoniae* [140].

According to various epidemiological reports from different international settings (based mainly on previously available EUCAST MICs and considering ECOFFs), resistance to fosfomycin is rare in MSSA (<5%), whereas it can be found in 5–30% of MRSA [16,141,142,143]. CONS are more frequently resistant to fosfomycin, but overall susceptibility is preserved in approximately 75% of isolates [16,132] In *S. pneumoniae*, the absence of validated breakpoints and ECOFF makes the epidemiological data difficult to interpret. However, penicillin-resistant *S. pneumoniae* was reported to have low MICs in 87% of isolates [144]. In VRE, susceptibility to fosfomycin can be found in 30% of isolates [144].

The risk of the emergence of resistance during fosfomycin monotherapy has been well established in vitro, although the extent of this phenomenon and its clinical consequences are still a matter of debate [145]. Grabein et al. [19] performed a systematic review and meta-analysis in 2016 on different topics related to fosfomycin use, including the emergence of resistance during monotherapy. They included 14 studies addressing this issue in both Gram-negative and Gram-positive bacteria. The highest reported incidence of emergence of resistance was 17.9%, but the pooled estimate across the 14 studies was 3.4% [19]. The emergence of resistance seems to be more frequent in Gram-negative isolates. A fitness cost for mutant bacteria could explain the discrepancy between the high risk of resistance development found in vitro and the data coming from in vivo studies [145].

Although the impact of the emergence of resistance on clinical outcomes is yet to be defined, fosfomycin has been used mainly in combination therapy, especially in previous years [19]. Combination therapy seems particularly preferable in the case of non-urinary infections and when devices or high inoculum are present. Historically, the companion drug in anti-Gram-positive treatments included beta-lactams, fluoroquinolones, glycopeptides, rifampin [14,19]. There is currently growing interest in using fosfomycin in combination with daptomycin when treating staphylococcal BSI, IE and intracardiac device associated infections. Another promising association is fosfomycin plus linezolid for the rescue therapy of severe VAP and CNS infections. These combinations may provide a synergistic antibacterial activity and reduce the risk of the emergence of resistance [15,19,146].

## 2. Conclusions

In conclusion, antibiotic resistance to molecules commonly prescribed for the treatment of antibiotic-resistant Gram-positive pathogens is overall infrequent, but clinically relevant. Breakpoints are often not validated because of insufficient evidence, making the interpretation of susceptibility tests and epidemiological data difficult. For some antibiotics, particularly daptomycin and fosfomycin, there is a risk of the emergence of resistance during treatment when they are prescribed as monotherapy for difficult-to-treat infections; therefore, appropriate combination therapy can be necessary. The clinician must be aware of the existence of these resistant strains and their clinical implications, requesting susceptibility testing when appropriate and judiciously choosing between monotherapy and combination therapy. Antimicrobial stewardship, epidemiological surveillance, and infection control measures are essential to preserving the activity of these precious antibiotics.

## 3. Future Directions

Many aspects of antibiotic resistance toward molecules treating resistant Gram-positive pathogens still need to be better clarified. More evidence is needed in order to inform reliable ECOFFs and MICs, which in many cases are not yet available. The epidemiology of resistance is a dynamic phenomenon, needing continuous monitoring; furthermore, more data are needed concerning the situation in low-income settings. Resistance mechanisms are not fully understood and deserve further studies. Frequency and risk factors for the emergence of resistance during treatment, as well as the clinical impact of this phenomenon, have to be better defined. The role of combination therapies used to improve bactericidal activity and prevent resistance is the subject of huge debate and will probably be a hot topic during the coming years.

## Figures and Tables

**Table 1:** Principal indications of molecules commonly prescribed for the treatment of antibiotic-resistant Gram-positive pathogens.

Antibiotic	Officially Licensed Indications	Other Common Off-Label Uses
Daptomycin	- ABSSSI - *S. aureus* BSI - *S. aureus* right-sided IE	- Left-sided IE - Intravascular device-associated infections - Osteoarticular infections, including PJI - VRE infections
Dalbavancin	- ABSSSI	- IE - Intravascular device-associated infections - Osteoarticular infections, including PJI
Linezolid	- ABSSSI - CAP - HAP	- CNS infections - VAP - IAI - Osteoarticular infections, including PJI - VRE infections - Nocardiodis - Drug-resistant tuberculosis
Tedizolid	- ABSSSI	- Similar to linezolid, with much less clinical experience
Ceftaroline	- ABSSSI - CAP	- BSI - IE - Intravascular device-associated infections - HAP/VAP - Osteoarticular infections, including PJI
Ceftobiprole ^1^	- CAP - HAP	- Similar to ceftaroline, with less clinical experience
Fosfomycin (IV use)	- ABSSSI - Complicated UTI - Complicated IAI - IE - HAP/VAP - Osteoarticular infections, including PJI - CNS infections	- Intravascular device-associated infections

ABSSSI: acute bacterial skin and skin structure infections; BSI: blood stream infections; CAP: community-acquired pneumonia; CoNS: coagulase-negative staphylococci; CNS: central nervous system; HAP: hospital-acquired pneumonia; IAI: intra-abdominal infections; IE: infective endocarditis; PJI: prosthetic joint infections; UTI: urinary tract infections; VAP: ventilator-associated pneumonia; VRE: vancomycin-resistant *Enterococcus faecium*. ^1^: approved in European Union, Canada and Switzerland.

**Table 2:** Breakpoints for major Gram-positive bacteria according to the European Committee on Antimicrobial Susceptibility Testing (EUCAST) and the Clinical and Laboratory Standards Institute (CLSI).

Pathogen	Antibiotic	EUCAST Version 14.0 (mg/L) [31]		CLSI M100 Version 2023 (mg/L) [32]	
		S≤	R>	S≤	R>
*Staphylococcus aureus* and coagulase-negative staphylococci (CoNS)	Daptomycin	1	1	1	1
	Dalbavancin	0.125	0.125	0.25 ^1^	0.25 ^1^
	Linezolid	4	4	4	8
	Tedizolid	0.5	0.5	0.5 ^1^	2 ^1^
	Ceftaroline	1 ^1^	2 ^1,2^	1 ^1^	8 ^1^
	Ceftobiprole	2	2	-	-
	Fosfomycin	-	-	-	-
*Enterococcus* spp.	Daptomycin	IE	IE	2 ^3^	8
	Dalbavancin	IE	IE	0.25 ^4^	0.25 ^4^
	Linezolid	4	4	2	4
	Tedizolid	IE	IE	0.5	0.5 ^4^
	Ceftaroline	-	-	-	-
	Ceftobiprole	-	-	-	-
	Fosfomycin	-	-	64	256 ^4^
Streptococcus groups A, B, C, and G	Daptomycin	1	1	1	1
	Dalbavancin	0.125	0.125	0.25	0.25
	Linezolid	2	2	2	2
	Tedizolid	0.5	0.5	0.5 ^5^	0.5 ^5^
	Ceftaroline	¥	¥	0.5	0.5
	Ceftobiprole	IE	IE	-	-
	Fosfomycin	-	-	-	-
*Streptococcus pneumoniae*	Daptomycin	IE	IE	-	-
	Dalbavancin	IE	IE	-	-
	Linezolid	2	2	2	2
	Tedizolid	IE	IE	-	-
	Ceftaroline	0.25	0.25	0.5	0.5
	Ceftobiprole	0.5	0.5	-	-
	Fosfomycin	-	-	-	-
Viridans group streptococci	Daptomycin	-	-	1	1
	Dalbavancin	0.125	0.125	0.25 ^6^	0.25 ^6^
	Linezolid	IE	IE	2	2
	Tedizolid	0.5	0.5	-	-
	Ceftaroline	-	-	-	-
	Ceftobiprole	-	-	-	-
	Fosfomycin	-	-	-	-

^1^: Staphylococcus aureus only. ^2^: For pneumonia, the breakpoint for resistance is >1 mg/L. ^3^: Only the susceptible dose-dependent (SDD) category is provided for *Enterococcus faecium*, with an MIC ≤ 4 mg/L. ^4^: Enterococcus faecalis only. ^5^: Streptococcus pyogenes and Streptococcus agalactiae only. ^6^: Streptococcus anginosus only. ¥: Inferred from bencylpenicillin susceptibility.
